# The spatio-temporal features of chicken mitochondrial ND2 gene heteroplasmy and the effects of nutrition factors on this gene

**DOI:** 10.1038/s41598-020-59703-y

**Published:** 2020-02-19

**Authors:** Suliang Yang, Yangyang Huo, Huanjie Wang, Jiefei Ji, Wen Chen, Yanqun Huang

**Affiliations:** College of Livestock Husbandry and Veterinary Engineering, Henan Agricultural University, No. 15 Longzi Lake University Campus, Zhengzhou, 450046 P.R. China

**Keywords:** Animal breeding, Gene amplification, Sequencing

## Abstract

Mitochondrial heterogeneity is the presence of two or more types of mitochondrial (mt)DNA in the same individual/tissue/cell. It is closely related to animal health and disease. *ND2* is a protein-coding gene in mtDNA, which participates in mitochondrial respiratory chain and oxidative phosphorylation. In previous studies, we observed that the mt.A5703T and mt.T5727G sites in the *ND2* gene were the heteroplasmic variation sites. We used pyrophosphate sequencing technology to examine chicken mt.A5703T and mt.T5727G heteroplasmic sites in the *ND2* gene, in different tissues and at different development stages in chickens. We also investigated whether nutritional factors could affect the mt.A5703T and mt.T5727G heteroplasmy. Our results showed that chicken mt.A5703T and mt.T5727G heteroplasmy had clear spatio-temporal specificities, which varied between tissues/development stages. The mtDNA heterogeneity was relatively stable upon nutrition intervention, 30% dietary energy restriction (from 18 to 48 days old) and different types of dietary fats (at 5% concentration, from 1 to 42 days old) did not change the breast muscle heteroplasmy of broilers at the mt.A5703T and mt.T5727G sites. In addition, multiple potential heteroplasmic sites were detected by clone sequencing in the *ND2* region, which potentially reflected abundant heteroplasmy in the chicken mitochondrial genome. These results provide an important reference for further research on heteroplasmy in chicken mitochondria.

## Introduction

Mitochondria play key physiological roles in the complex relationships between nutrition, health^1^ and diseases^2^. Vertebrate mitochondrial DNA (mtDNA) is a nude ring molecule lacking histone protections, which causes mitochondria to be prone to high variation frequencies and sequence diversity^3,4^. Heteroplasmy is the presence of more than one type of mtDNA in the mitochondria of a single individual, it is divided into length heterogeneity and point heterogeneity. The former refers to the insertion and deletion of nucleotide fragments^5,6^, whereas the latter refers to two or more bases^7,8^. It has been reported that mitochondrial heterogeneity is common in humans^9^, mitochondrial heterogeneity is the primary inheritance mode of mitochondrial disease, several cancers and aging^10–12^. By applying the population-genetic framework for modeling mitochondrial heteroplasmy to previously published heteroplasmy frequency data, Wilton, *et al*.^13^ demonstrated a severe effective germline bottleneck, comprised of a cumulative genetic drift occurring between the divergence of germline and somatic cells in the mother and the separation of germ layers in the offspring. However, the study of animal mitochondria is still in its infancy. As an important economic bird, few reports have been published on mitochondrial heterogeneity in chickens and there has been a lack of studies on the effects of nutritional factors on mtDNA heterogeneity in chickens.

The *ND2* gene is a mtDNA protein coding gene, involved in energy metabolism^14–18^. The gene product is extensively expressed in 15 tissues including the heart of hybrid chickens and its expression is affected by dietary fat types and age^19^. The mt.A5703T and mt.T5727G sites (refer to GenBank: AP003317) in the *mtND2* gene have been detected as heteroplasmic sites, using the polymerase chain reaction (PCR)-restriction fragment length polymorphism (RFLP) method^20^. It was found that *mtND2* heteroplasmic variations had significant effects on chicken growth traits, carcass parameters and meat quality, demonstrating the potential importance of related variation^20^. However, a study on the heterogeneity of these sites in term of nutritional factors, has not yet been reported in chickens.

Here we explored the mitochondrial heterogeneity in different chicken tissues and at various individual development stages, and studied the effects of dietary energy restriction and different types of dietary fats on heteroplasmy in chicken *mtND2* with pyrosequencing™ technology. Pyrosequencing is a real-time sequencing method for the analysis of short to medium length DNA sequences^21^. In addition, clone sequencing was used to identify mitochondrial variation variation/heterogeneity. It has been observed that mtDNA frequently inserts into the nuclear genome and forms mitochondrial pseudogenes. However in the above case, these sequences were non-functional, and therefore did not express^22^. In this study, we used cDNA as a template to exclude interference of mitochondrial pseudogenes. In addition, high fidelity Taq was used for variation detection. This enzyme has 3′ to 5′ exonuclease activity, and can prevent mispriming and enhance PCR specificity.

## Methods

### Experiment population and RNA sample extraction

Breast muscle from Dwarf Silky (DS) fowls was collected at embryonic day (E)12, E14, and E17 (three replicates per embryo stage) as well as one day and seven days after hatching (D1 and D7 respectively, three replicates per day stage). The other chicken embryos from the same period had been hatched and received normal feeding and management at the poultry resource farm (Zhengzhou city, Henan province, China). DS fowls fed and drank freely in a 23-h light and 1-h dark cycle environment. Basal diets comprised 16.5% crude protein and 11.6 MJ/kg metabolizable energy (ME), according to United States National Research Council (NRC) nutritional standards of chicken^23^ (1994). In addition, eleven tissue samples including leg muscle, cerebrum, cerebellum, lung, liver, bursa of fabricii, heart, intestine, kidney, muscular stomach, and glandular stomach were collected from seven day old DS fowl (three replicates). Approximately 10 g of tissue samples were snap-frozen in liquid nitrogen and stored at −80 °C for RNA extraction.

Breast muscle, from the energy restriction population, was used to study the effects of 30% dietary energy restriction on heteroplasmy in *mtND2*. Energy restriction populations were constructed as described in Wang, *et al*.^24^ (For details, see Supplementary Table 1). Fifty healthy female Arbor Acre (AA) commercial broilers of similar weights (average ±30 g) were selected at 18 days old and randomly allocated to the *ad libitum* group (AL; n = 25) and the energy restriction group (ER; n = 25). These broilers were tagged and housed individually in stainless steel cages in an environmentally controlled room, with 23 h illumination. From 18 to 48 days old, AL broilers were fed *ad libitum* with a control diet (13.17 MJ/kg ME) according to recommendations of the NRC nutritional standards of chicken^23^ (1994). Each ER broiler was subjected to 30% energy restriction. 30% energy restriction was achieved by cut down the metabolizable energy level of 10% dietary and reducing the 20% feed intakes. Except metabolizable energy, the supply of other nutrients for ER broilers were the same as that of AL broilers. Water was provided freely. Routine immunization procedures were used throughout the study. Twenty broilers of similar body weight were selected from each of the two groups and slaughtered at 48 days. Approximately 10 g of breast muscle tissue was snap-frozen in liquid nitrogen and stored at −80 °C for RNA extraction.

Breast muscle, from the chicken fat population, was used to study the effects of different types of dietary fats on heteroplasmy in *mtND2*. Chicken fat populations were constructed as described by Zhang, *et al*.^19^ (For details, see Supplementary Table 2). Specifically, 120 female Cobb-500 broilers of similar weights (average ±4 g) at 1 day old were randomly assigned to four groups: 5% linseed oil (LO); 5% sesame oil (SO); 5% lard grease (LG); and 5% corn oil (CO), with six replicates per group and five broilers per replicate. Each repeated broilers were raised in single cage. Broilers were fed according to the nutrient requirements of white-feathered broiler chickens^25^ (China, NY/T 33-2004). The fat treatment groups contained above four types of dietary oils at 5% concentration based on the basic diets. Chickens were tagged and housed in an environmentally controlled room, with 23 h illumination. Feed and water were provided freely. Routine immunization procedures were used throughout the study. From these groups,12 broilers were randomly selected (three birds per replicate) and slaughtered at 42 days. Approximately 10 g breast muscle was snap-frozen in liquid nitrogen and stored at −80 °C for RNA extraction.

All chickens received excellent care as outlined in the Guide for the Care and Use of Agricultural Animals in Research and Teaching (2010).

### RNA extraction and synthesis of cDNAs

Total RNA was extracted from 100 mg tissue using RNAiso Plus (TaKaRa, Dalian, China), according to the instruction manual. Isolated RNA was quantified by a Microspectrophotometer GeneQuant pro (Amersham Pharmacia Ltd, Bucks, UK) and agarose gel electrophoresis. Total RNA was used to synthesize cDNA using reverse transcription reagents (PrimeScript^RT^ reagent kit with gDNA Eraser, TaKaRa, Dalian, China) according to the instruction manual. The first strand cDNA samples were stored at −20 °C for later use. The quality of reverse transcribed cDNA was assessed by PCR using β-actin primers (F: 5′ACCGCAAATGCTTCTAAC3′; R: 5′CCAATCTCGTCTTGTTTTATG3′). The PCR product size was 93 bp. PCR amplifications were performed in a total volume of 12.5 μL, containing 6.5 μL Universal PCR Master mix, 1.0 μL cDNA (50 ng/μL), 0.5 μL forward and reverse primer, and made up to volume with distilled water. PCR parameters were: 5 min at 94 °C for pre-degeneration, then 30 cycles at 94 °C for 30 s, 60 °C for 30 s, 72 °C for 30 s and a 10 min extension at 72 °C.

### Construction of plasmids containing mt.A5703T and mt.T5727G sites

Based on a previous study in our laboratory^20^, six breast muscle samples from Gushi chicken (Henan native chicken) containing mt.A5703T and mt.T5727G variants were selected. RNA was extracted, and synthesized cDNA was used for cloning and sequencing. Briefly, ND2-1 primers (F: 5′ATCAGCCCTAATCCTCTTCTC3′; R: 5′GTGGCTATTGGGGTTATTTCT3′) were designed (Sangon Biotech, Shanghai, China) to amplify sequences containing the two variation sites. The PCR product was 642 bp. The PCR amplification was performed in a volume of 50 μL, containing 2.0 μL cDNA (50 ng/μL), 1.0 μL of forward and reverse primer (10 pm/μL) and 25 μL high fidelity Taq enzyme (PrimeSTAR^®^ Max DNA Polymerase, TaKaRa, Dalian, China). The parameters were: 10 min at 94 °C for pre-degeneration, followed by 32 cycles at 94 °C for 35 s, 60 °C for 35 s, 72 °C for 1 min and a 10 min extension at 72 °C. Purified PCR products were ligated into a pMD18-T vector according to kit instructions (TaKaRa, Dalian, China). Ten clones were prepared and sequenced, and the haplotypes were analyzed. DNAMAN (6.0.3) software was used for amino acid homology comparisons to predict amino acid changes on account of vertebrae mitochondrial genetic code. Haplotype analysis was performed using DNASP.5. Plasmids containing 5703A-5727T/5703T-5727G haplotypes (GenBank Accession No. AP003317) were used as positive controls for pyrosequencing.

### Heteroplasmy detection of mt.A5703T and mt.T5727G by pyrosequencing

To exclude the interference of mitochondrial pseudogenes, cDNA samples were used to identify heteroplasmy of mt.A5703T and mt.T5727G by pyrosequencing (For details, see Supplementary Method). Briefly, primers (F: 5′CCTCCTCCTAACTCACAGTCTCTTAA3′; R: 5′ biotin-AGAAGGCTAGGATTTTTCGTGTTTGT3′) were designed to amplify the area containing the mt.A5703T and mt.T5727G variations. The PCR reaction consisted of 4 μL 10× PCR buffer, 3.2 μL 2.5 mM dNTPs, 0.4 μL 10 μM forward and reverse primers, 2.5 U of Taq DNA polymerase (Takara Co, Dalian City, China), 2 μL bisulfite treated cDNA and distilled water to a final volume of 40 μL. PCR amplifications were performed using a standard PCR program, starting with 3 min at 95 °C for pre-degeneration, followed by 45 cycles for 15 s at 95 °C, 20 s at 56 °C for annealing, 30 s at 72 °C and a 5 min extension at 72 °C. The purified single-strand products were mixed with 40 μL sequencing buffer (including 0.5 μM Pyro-sequencing primer, 5′CCATTCAGCCTCCGA3′). After two min denaturation at 80 °C, sequencing was conducted on a PyroMark Q96 ID sequencer (Qiagen, Germany). All steps were performed according to manufacturer’s protocols. Plasmids containing the 5703A-5727T and 5703T-5727G haplotype (GenBank Accession No.AP003317) were used as positive controls. The relative percentages of allele at mt.A5703T and mt.T5727G sites were scored by analyzing the corresponding variation sites. Each cDNA sample was measured in triplicate.

### Statistical analysis

Data were analyzed using the statistics program SPSS (version 19.0, SPSS Inc., Chicago, IL, USA). One-way analysis of variance (ANOVA) was conducted to assess whether the four types of dietary fats or the 30% dietary energy restriction affected heteroplasmy of the mtND2 gene. The Duncan method was used for multiple comparisons. P <0.05 was considered statistically significant.

### Ethics approval

This study was approved by the Animal Care and Use Committee of Henan Agricultural University.

## Results

### Variation sites and haplotypes

Referring to the research on two heteroplasmic sites (mt.A5703T and mt.T5727G) in our previous work^20^, six individuals were selected for clone sequencing with *ND2-1* primer set. Approximately 29 variation sites were detected from the sequencing samples, 16 variants were predicted to cause amino acid changes (Table 1).

**Table 1:** Variants in the mtND2 gene and predicted amino acid changes.

Variation sites	Nucleotide change	Amino acid change
T5484C	TGA → CGA	W → R
C5509T	ACA → ATA	T → M
C5523A	CCG → ACG	P → T
A5559G	ATC → GTC	I → V
A5562G	AAA → GAA	K → E
T5586C	TTC → CTC	F → L
T5613C	TCC → CCC	S → P
T5674C	CTC → CCC	L → P
T5685C	TCA → CCA	S → P
A5703T	ACC → TCC	T → S
T5727G	CTA → TTA	S → A
T5758C	CTA → CCA	L → P
A5764G	CAA → CGA	Q → R
T5791C	TTC → TCC	F → S
T5796C	TCC → CCC	S → P
A5823G	ATA → GTA	M → V

Twenty-nine variation sites constructed 21 haplotypes (Table 2). The major haplotypes were H1, H2 and H16, the rest were unique. H1 (wild haplotype) occurred most frequently and was present in samples five and six. H2 was a shared haplotype in samples one, two and three. H16 was found only in sample four. The three sites including A5703T, T5727G, G5963A were found to be in complete linkage disequilibrium in all samples. Each sample had at least two haplotypes, which showed that each was a potentially heteroplasmic individual.

**Table 2:** The haplotype distribution of the mtND2 gene for six samples (by clone sequencing)^a^.

Haplotype	55555555555555555555555555566 55566666777777777888889999900 56815678023556699123364678800 92636345376384916432596382626	Number	Sample one	Sample two	Sample three	Sample four	Sample five	Sample six
Wild type	AATTTATTATCGTACTTTATTTTATATAG							
H1	----------------------------------------------------	17					8	9
H2	--------------TG------------------------G--------	14	1	9	4			
H3	--------------TG----------C---G-------G--------	1	1					
H4	--------C----TG------------------------G---------	1	1					
H5	--------------TG----C------------------G---------	1	1					
H6	---------C---TG------------C----------G---------	1	1					
H7	--------------TG------G----------------G---------	1	1					
H8	------C------TG------------------------G---------	1	1					
H9	--------------TG------------------------GC--------	1		1				
H10	------------CTG------------------------G---------	1			1			
H11	----C--------TG------------------------G---------	1			1			
H12	--------------TG------------------------G------G-	1			1			
H13	---------CC-TG-------T--------------- G---------	1			1			
H14	--G----------TG------------------------G---------	1			1			
H15	G------------TGT---------------------- G---------	1			1			
H16	------------------------------------C--------------	7				7		
H17	-------------------------------------C-C-----------	1				1		
H18	-------------------------------------C--------A----	1				1		
H19	--------------------T----------C--------------------	1					1	
H20	---------------------------------------------------A	1					1	
H21	-----------------------------------C---------G------	1						1
Total			7	2	7	3	3	2

^a^Standard reference sources of *mtND2* gene sequence: GenBank (AP003317), no involving the whole genome sequence.

### Positive controls for pyrosequencing containing mt.A5703T and mt.T5727G variation

**Figure 1:**
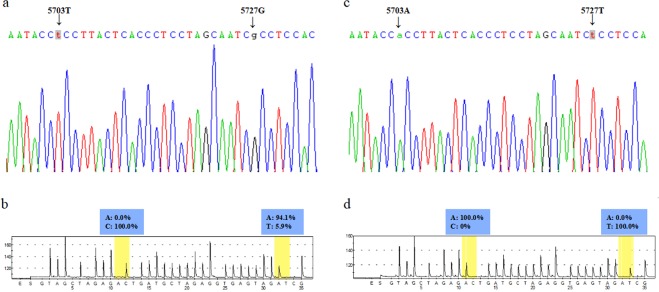
Positive control plasmid for pyrosequencing. The Sanger sequencing peak map (**a**) and the pyrosequencing peak map in complement strand (**b**) for the positive control clone that contains mt.5703T and mt.5727G alleles. The Sanger sequencing peak map (**c**) and the pyrosequencing peak map in complement strand (**d**) for the positive control clone that contains mt.5703 A and mt.5727 T alleles.

Two plasmids, containing mt.5703T - mt.5727G sequencing haplotype (plasmid I, Fig. 1a) and mt.5703 A - mt.5727 T haplotype (plasmid II, Fig. 1c) were selected as positive controls for pyrophosphate sequencing. It was observed that the mt.T5727G site had 100% G alleles in plasmid I (Fig. 1b) and 100% T alleles in plasmid II (Fig. 1d). At the mt.A5703T site, the percentage of T alleles in plasmid I was 94.1% (Fig. 1b), and the percentage of A alleles in plasmid II was 100% (Fig. 1d). For pyrosequencing, all data at the mt.A5703T site was eliminated background values, and then analyzed further.

### The spatio-temporal features of heteroplasmy at mt.T5727G and mt.A5703T sites

We investigated the heteroplasmic features of mt.T5727G and mt.A5703T sites in chicken breast tissues, at embryonic and post-hatching stages (Table 3). In the embryonic stage, no heterogeneity was detected in chicken breast tissues from E12 and E14, while one of three E17 individuals presented clear heterogeneity. In this sample, the G allele was 27.9% for the mt.T5727G site and the T allele was 25.6% for the mt.A5703T site. At post-hatching, no heterogenic individuals were detected at the D1 stage, while one of three stage D7 individuals (D7-1) showed weak heterogeneity at the mt.T5727G site (the G allele was 96.9%) and the mt.A5703T site (the T allele was 93.0%).

**Table 3:** The heteroplasmic sites, mt.T5727G and mt.A5703T, in chicken breast muscle from different development periods.

Periods	mt.T5727G		mt.A5703T	
	Allele T (%)	Allele G (%)	Allele A (%)	Allele T (%)
E12 (n = 3)	100.0	0.0	100.0	0.0
E14 (n = 3)	100.0	0.0	100.0	0.0
E17 (n = 1)	72.2	27.9	74.4	25.6
E17 (n = 2)	100.0	0.0	100.0	0.0
D1 (n = 3)	100.0	0.0	100.0	0.0
D7 (n = 2)	100.0	0.0	100.0	0.0
D7 (n = 1)	3.1	96.9	7.0	93.0

To determine tissue heteroplasmic features of mt.T5727G and mt.A5703T sites, twelve tissue samples from D7-1 were selected for further pyrophosphate sequencing. These data showed (Table 4) that heteroplasmy at the mt.T5727G and mt.A5703T sites had clear tissue specificity. Firstly, 12 tissue samples from D7-1 had different predominant alleles; the predominant allele was T for the mt.T5727G site and A for the mt.A5703T site in cerebrum, cerebellum, intestines, kidney, lung, liver, glandular stomach, muscular stomach and heart, while the predominant allele was G for the mt.T5727G site and T for the mt.A5703T site in breast muscle, leg muscle and bursa of fabricii. Secondly, different tissues showed different degrees of heterogeneity. No alternative alleles (mt.5727G and mt.5703T) were detected in cerebrum, cerebellum, intestines, kidney, lung, liver, glandular stomach, muscular stomach and heart for both the mt.T5727G and mt.A5703T sites. Breast and leg muscle showed similar weak heterogeneity; the G allele at the mt.T5727G site was 96.9% for breast muscle and 96.4% for leg muscle. The bursa of fabricii showed high heterogeneity at the mt.T5727G site (the G allele was 54.2%) and the mt.A5703T site (the T allele was 47.8%).

**Table 4:** The heteroplasmic sites, mt.T5727G and mt.A5703T, in different tissues from the same individuals.

Sample	mt.T5727G		mt.A5703T	
	Allele T (%)	Allele G (%)	Allele A (%)	Allele T (%)
Breast muscle	3.1	96.9	7.0	93.0
Leg muscle	3.6	96.4	5.2	94.8
Bursa of fabricii	45.8	54.2	52.2	47.8
Cerebrum	100.0	0.0	100.0	0.0
Cerebellum	100.0	0.0	100.0	0.0
Intestines	100.0	0.0	100.0	0.0
Kidney	100.0	0.0	100.0	0.0
Lung	100.0	0.0	100.0	0.0
Liver	100.0	0.0	100.0	0.0
Glandular stomach	100.0	0.0	100.0	0.0
Muscular stomach	100.0	0.0	100.0	0.0
Heart	100.0	0.0	100.0	0.0

In addition, it was observed that no matter in the heterogeneity or non-heterogeneity individuals/tissues, the mt.T5727G and mt.A5703T sites had similar predominant alleles and predominant allele frequencies which showed they were in strong linkage disequilibrium.

### The effects of 30% dietary energy restriction on mitochondrial ND2 gene heterogeneity

**Figure 2:**
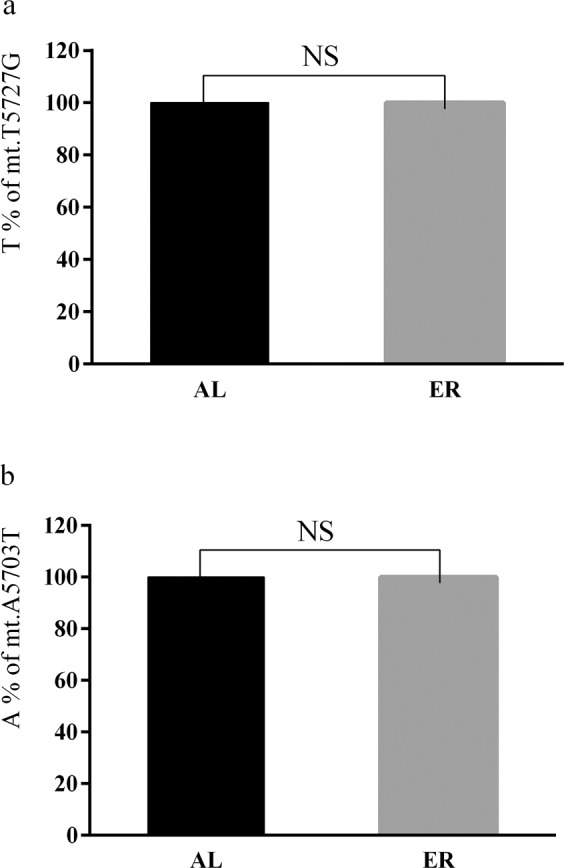
Comparisons of predominant allele frequency for mt.T5727G and mt.A5703T sites between 30% energy restriction group (ER) and *ad libitum* group (AL). (**a**) mt.T5727G site; (**b**) mt.A5703T site. n = 20 for each group. *NS* means no significance.

We used pyrophosphate sequencing to examine the heteroplasmy of broiler breast muscles on 30% dietary energy restriction and to determine the mt.T5727G and mt.A5703T sites in the AL group and ER group. The results showed (Fig. 2) that both ER and AL groups were TT homozygous (the T allele was 100%) at the mt.T5727G site. There was no heterogeneity for the mt.T5727G site. At the mt.A5703T site, both ER and AL groups were AA homozygous (the A allele was 100%). There was also no heterogeneity for the mt.A5703T site. There were no allele frequency differences between the ER and AL groups for the mt.T5727G and mt.A5703T sites. This data indicated that a 30% energy dietary restriction did not cause heterogeneity changes at mt.T5727G and mt.A5703T sites.

### Effects of different types of dietary fats on mitochondrial ND2 gene heterogeneity

**Figure 3:**
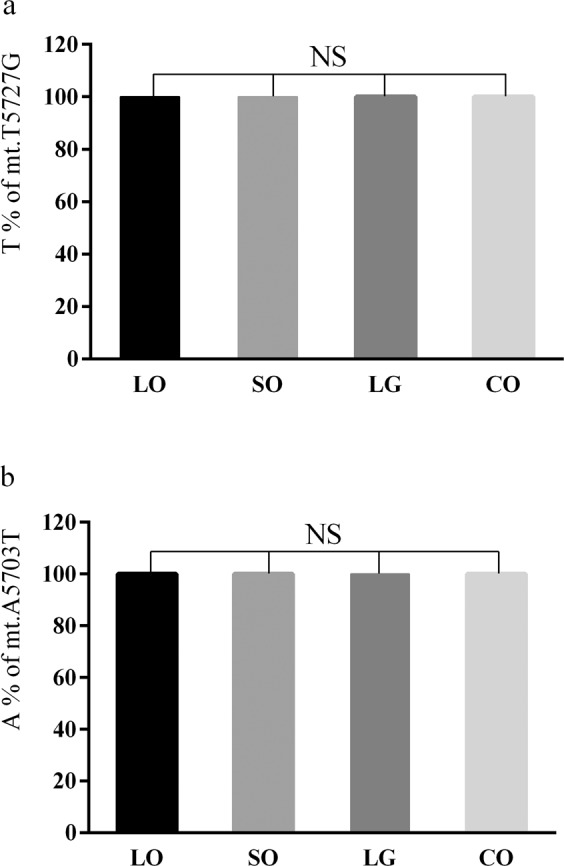
Comparisons of predominant allele frequency for the mt.T5727G and mt.A5703T sites among different fat treatment group. (**a**) The mt.T5727G site; (**b**) the mt.A5703T site. LO, 5% linseed oil group; SO, 5% sesame oil group; LG, 5% lard grease group; CO, 5% corn oil group. n = 5 for each group. *NS* means no significance.

We used pyrophosphate sequencing to examine mt.T5727G and mt.A5703T heteroplasmy in broilers fed LO; LG; SO; and CO. The results showed (Fig. 3) that the four oil groups were TT homozygous (the T allele was 100%) for the mt.T5727G site, whereas no heterogeneity was detected at the mt.T5727G site. The four oil groups were AA homozygous (the A allele was 100%) at the mt.A5703T site, and no heterogeneity was found at the mt.A5703T site. There were no allele frequency differences among the different groups for the mt.T5727G and mt.A5703T sites. These data indicated that different types of dietary fats did not cause heterogeneity changes at mt.T5727G and mt.A5703T sites.

## Discussion

Mitochondrial *ND2* is an important subunit in the mitochondrial respiratory chain complex, and is involved oxidative phosphorylation^26–30^. This *mtND2* mutation is associated with Leigh Syndrome^31^. Near, *et al*.^32^ found that the *ND2* gene had a faster evolutionary rate than other mitochondrial protein coding and rRNA genes. In this study, we detected 29 potential heterogenic variants of the *ND2* gene by sequencing, constituting 21 haplotypes. The mt.T5727G and mt.A5703T sites in the chicken *ND2* gene were in strong linkage disequilibrium, no matter in the heterogeneous or non-heterogeneous individuals/tissues correspondingly. The mt.5727 T and mt.5703 A sites were the predominant alleles, and mt.5727T-mt.5703 A was the predominant haplotype.

The heterogeneity of mtDNA is associated with human health^33–35^. Li, *et al*.^21^ used Solexa high-throughput sequencing (Illumina Genome Analyzer) to identify 37 heteroplasmic sites at 10% frequencies or higher at 34 sites in 32 people, and 4,577 heteroplasmies (with an alternative allele frequency of at least 0.5%) at 393 positions across the human mtDNA genome^9^. He, *et al*.^36^ detected widespread heterogeneity in the mtDNA of normal human cells, the frequency of heteroplasmic variants varied considerably between different tissues in the same individual. Huang, *et al*.^37^ identified 178 cases of heteroplasmy in the chicken mitochondrial genome (at the 0.5% level). In our research, mt.T5727G and mt.A5703T heterogeneity in the *ND2* gene varied between tissues and different developing stages. Heteroplasmies in single tissues are more likely to be somatic mutations, whereas heteroplasmies in three or more tissues are more likely to be inherited (or occur early in development)^9^. Mitochondrial heteroplasmy is also strongly age-related. Sondheimer, *et al*.^38^ found that mitochondrial heteroplasmy across the human genome increased significantly with advanced age. This study showed that no heterogeneous individuals were found from the earlier embryonic development stages and the first day of post-hatch, which may be related with the age feature of mitochondrial heterogeneity.

Until now, there have been no reports on how nutritional factors impact mitochondrial heterogeneity. In this study, it was found that 30% energy dietary restriction, or 5% different types of dietary oil supplementation, did not affect heterogeneity of mt.T5727G and mt.A5703T sites in *mtND2*. These observations highlighted the relative stability of mitochondrial heterogeneity under nutritional compromise. It was observed chicken heteroplasmy decreased greatly from the F0 to F1 generations at mt.A5703T and mt.T5727G sites (or mt.A5694T and mt.T5718G site, refer to GeneBank: NC_001323.1)^37^. Sharpley *et al*. reported that the admixture of two normal but different mouse mtDNAs can be genetically unstable and can produce adverse physiological effects such as reducing activity and food intake, which may explain the advantage of uniparental inheritance of mtDNA^39^.

It has been reported that energy restriction mitigates some detrimental effects of aging, and prolongs lifespan^40^. Energy restriction has been shown to reduce reactive oxygen species (ROS) production, mitochondrial function, biosynthesis and respiration, suggesting that energy restriction affects mitochondrial related functions^41^. In our previous study, a 30% energy restriction significantly reduced the expression of mitochondrial *ND1* gene in broilers’ liver^24^. Energy restriction preserves mitochondrial function by protecting the integrity and function of cellular components, rather than increasing mitochondrial biogenesis^40^.

Sealls, *et al*.^42^ reported that when fed 6% lard, rapeseed oil and fish oil, rats increased the expression of lipid producing proteins. Hynes, *et al*.^43^ found that dietary vegetable and fish oils significantly increased the expression of visceral obesity gene mRNA in rats. Rodríguez, *et al*.^44^ also showed that olive oil and sunflower oil increased the expression of obesity genes in rats. It is believed the type of dietary fats affects mitochondrion structure^45,46^.

## Conclusion

Our data showed that the heterogeneity of the mt.T5727G and mt.A5703T sites had clear spatio-temporal specificities. The heterogeneity of the mt.T5727G and mt.A5703T sites varied greatly in different tissues of one seven-day old chicken and the heterogeneity types included different predominant alleles and different predominant allele frequencies in tissues. Moreover, the mt.T5727G and mt.A5703T sites were in high linkage disequilibrium in heterogeneous/non-heterogeneous samples. In conducting a study with 30% dietary energy restriction (from 18–48 day old) and a study of chicken dietary supplemented with 5% LO; 5% LG; 5% SO; and 5% CO (from 1–42 day old), we did not observe any heterogeneity effects in broiler breast muscle at mt.T5727G and mt.A5703T sites.

## Supplementary information

Supplementary Information.

## Data Availability

The datasets used and analyzed in this study are available from the corresponding author, upon request.
