# Supplementary material for: The spatio-temporal features of chicken mitochondrial ND2 gene heteroplasmy and the effects of nutrition factors on this gene
Source: Sci Rep. 2020 Feb 19;10:2972. doi: 10.1038/s41598-020-59703-y (PMC7031424; doi:10.1038/s41598-020-59703-y)

The spatio-temporal features of chicken mitochondrial ND2 gene heteroplasmy and the effects of nutrition factors on this gene

Suliang Yang[1], Yangyang Huo[1], Huanjie Wang[1], Jiefei Ji[1], Wen Chen[1] and Yanqun Huang[*1]

---

[1] College of Livestock Husbandry and Veterinary Engineering, Henan Agricultural University, No. 15 Longzi Lake University Campus, Zhengzhou, 450046, P. R. China

[*] Corresponding author

Yanqun Huang

Postal address: College of Livestock Husbandry and Veterinary Engineering, Henan Agricultural University, No. 15 Longzi Lake University Campus, Zhengzhou, 450046, P. R. China

Tel: +8613838074456

E-mail: hyanqun@aliyun.com

Table S1 *The ingredients and nutrient levels of energy restriction experimental diets (air-dry basis) %*

Items	AL [a)]	ER [a)]
Ingredients		
Corn	57.94	48.74
Soybean oil	6.65	1.7
Soybean meal	31.1	44.23
$CaCO_3$	1.33	1.63
$CaHPO_4$	1.34	1.74
Choline Chloride	0.1	0.13
L-Lysine	0.08	
Methionine	0.11	0.15
Salt	0.35	0.43
Premix [b)]	1.00	1.25
Sum	100	100
Nutrient levels [c)]		
ME /(MJ /kg)	13.17	11.53
CP	19.00	23.75
Ca	0.90	1.13
TP	0.55	0.68
NPP	0.35	0.44
Lys	1.05	1.31
Met	0.40	0.50
CF	8.97	4.04

[a)]AL for the ad libitum group and ER for the energy restriction group.

[b)]Premix ,Vitamin and mineral mix per kilogram of feed: vitamin A, 12 000IU; vitamin D_3, 2 400 IU; vitamin K_3, 3 mg; vitamin B_1, 2.2 mg; vitamin B_2, 8 mg; vitamin B_6, 5 mg; vitamin B_{12}, 11μg; folic acid, 1.5 mg; biotin,150 μg; calcium pantothenate, 25 mg; nicotinic acid, 65 mg; Mn, 60 mg; Zn, 40 mg; I, 0.33 mg; Fe, 80 mg; Cu, 8 mg; Se, 0.15 mg.

[c)]Calculated in accordance with nutrient requirements of broilers of NRC nutritional standards of chicken (1994).

Table S2 *The ingredients and nutrient levels of different types of dietary fats experimental diets (air-dry basis) %*

Items	Aged from 1 to 3 weeks		Aged from 4 to 6 weeks	
	LO; SO; CO [a)]	LG [a)]	LO; SO; CO	LG
Ingredients				
Corn	51.15	50.65	57.35	56.94
Oil	5.00	5.00	5.00	5.00
Soybean meal	35.36	35.46	29.70	31.00
Fish meal	3.00	3.00	3.00	3.00
Corn gluten meal			0.90	
NaCl	0.35	0.35	0.35	0.35
Limestone	1.26	1.26	1.19	1.18
CaHPO 4	1.55	1.55	1.26	1.26
50% choline chloride	0.26	0.26	0.20	0.20
Lys	0.03	0.03		0.01
Met	0.14	0.14	0.05	0.06
Limestone	0.90	1.30		
Premix [b)]	1.00	1.00	1.00	1.00
Total	100.00	100.00	100.00	100.00
Nutrient levels [c)]				
ME /(MJ /kg)	12.56	12.56	12.98	12.98
CP	21.50	21.50	20.01	20.01
Ca	1.01	1.01	0.90	0.90
TP	0.68	0.68	0.62	0.62
AP	0.45	0.45	0.40	0.40
Lys	1.15	1.16	1.02	1.05
Met	0.50	0.50	0.40	0.41

[a)]LO for the linseed oil group, SO for the sesame oil group, CO for the corn oil group and LG for the lard grease group.

[b)]Premix provided the following per kg of diets: VA 0.45 mg, VD 3 0.005 mg, VE 10.00

mg, VK 0.50 mg, VB_1 1.80 mg, VB_2 3.60 mg, VB_6 3.50 mg, VB_{12} 0.002 5 mg, niacin acid 35.00 mg, folic acid 0.55 mg, biotin 0.20 mg, pantothenic acid 10.00 mg, Zn ($ZnSO_4 \cdot 7H_2O$) 40.00 mg, Mn ($MnSO_4 \cdot 5H2O$) 60.00 mg, Fe ($FeSO_4 \cdot 7H_2O$) 80.00 mg, Cu ($CuSO_4 \cdot 5H_2O$), 10.00 mg, I (KI) 0.35 mg, Se ($Na_2SeO_3 \cdot 5H_2O$) 0.15 mg.

[c)]Calculated in accordance with the nutrient requirements of white-feathered broiler chickens (NY/T 33-2004).

Supplementary Method: *Pyrophosphate sequencing technology*

Biotin labeled primers were used to amplify polymerase chain reaction (PCR). Then the PCR product was purified and transformed into single chain. DNA polymerase, ATP sulfatase, luciferase and ATP diphosphatase were added to the mixture. A type of dNTP was added each time during the sequencing process. If the dNTP could complement the template chain, a series of reactions occurred under the action of four enzymes. Finally, the fluorescence signal was converted into electrical signal, showing peaks of different heights, the height of which was proportional to the number of bases. On the contrary, when dNTP could not bind to the template chain, it would be directly degraded by ATP-diphosphatase, accordingly, there would be no peak map. The figure below showed the principle of pyrophosphate sequencing technology.

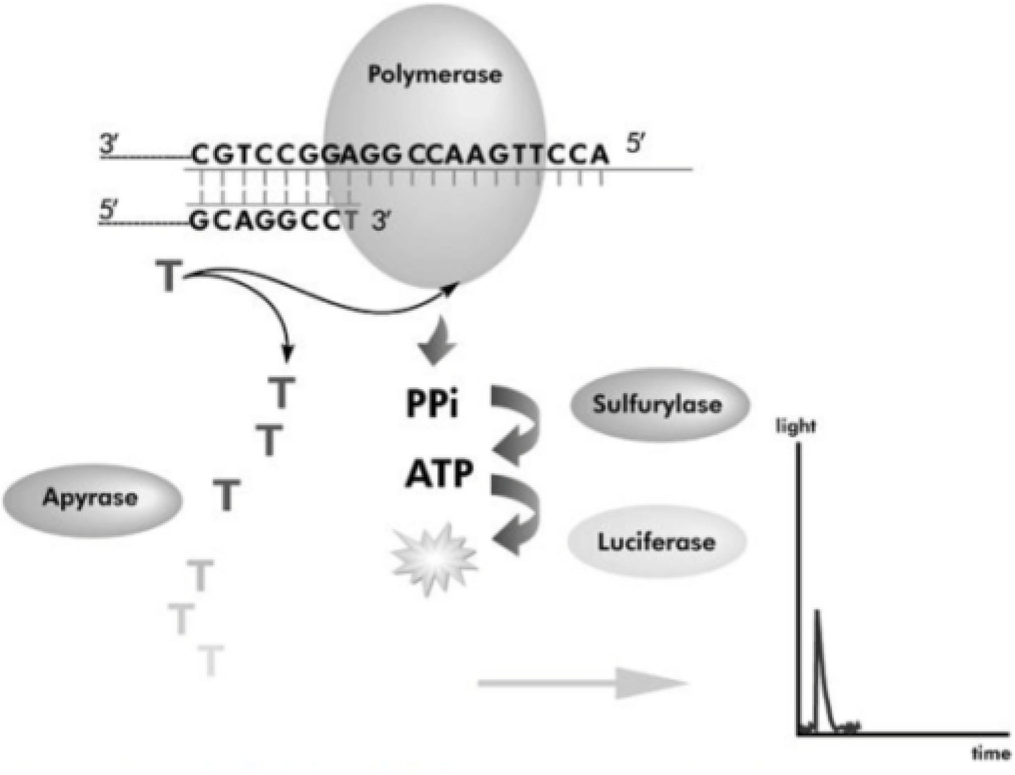

Supplement: Supplementary file 1 — Supplementary Information. [file 41598_2020_59703_MOESM1_ESM.pdf]
